# Successful recovery of infective endocarditis-induced rapidly progressive glomerulonephritis by steroid therapy combined with antibiotics: a case report

**DOI:** 10.1186/1471-2369-5-18

**Published:** 2004-12-21

**Authors:** Daisuke Koya, Kazuyuki Shibuya, Ryuichi Kikkawa, Masakazu Haneda

**Affiliations:** 1 Department of Medicine, Shiga University of Medical Science, Seta, Otsu, Shiga 520-2192, Japan; 2 Internal Medicine II, Asahikawa Medical College, Asahikawa, Hokkaido 078-8510, Japan

## Abstract

**Background:**

The mortality rate among patients with infective endocarditis, especially associated with the presence of complications or coexisting conditions such as renal failure and the use of combined medical and surgical therapy remains still high. Prolonged parenteral administration of a bactericidal antimicrobial agent or combination of agents is usually recommended, however, the optimal therapy for infective endocarditis associated with renal injury is not adequately defined.

**Case presentation:**

Patient was a 24-years old man who presented to our hospital with fever, fatigue, and rapidly progressive glomerulonephritis. He had a history of ventricular septum defect (VSD). A renal biopsy specimen revealed crescentic glomerulonephritis and echocardiogram revealed VSD with vegetation on the tricuspid valve. Specimens of blood demonstrated Propionibacterium Acnes. The intensive antibiotic therapy with penicillin G was started without clinical improvement of renal function or resolution of fever over the next 7 days. After the short-term treatment of low dose of corticosteroid combined with continuous antibiotics, high fever and renal insufficiency were dramatically improved.

**Conclusion:**

Although renal function in our case worsened despite therapy with antibiotics, a short-term and low dose of corticosteroid therapy with antibiotics was able to recover renal function and the patient finally underwent tricuspid valve-plasty and VSD closure. We suggest that the patients with rapidly progressive glomerulonephritis associated with infective endocarditis might be treated with a short-term and low dose of corticosteroid successfully.

## Background

Infective endocarditis has been classified as acute or subacute-chronic based on the clinical presentation and often presents extracardiac findings such as fever, anorexia, weight loss, malaise, and night sweats [1]. The prognosis of infective endocarditis has been shown to be strongly influenced by the complication of congestive heart failure and stroke [1]. Furthermore, glomerulonephritis, especially rapidly progressive glomerulonephritis, is also one of the complications associated with poor prognosis [2]. Infective endocarditis-induced rapidly progressive glomerulonephritis is treated with antibiotics alone, but it sometimes results in end-stage renal failure [2]. Although effective strategies to treat rapidly progressive glomerulonephritis have not been established, steroid therapy, immunosuppressive therapy, and plasmapheresis in addition to antibiotic therapy has been shown to be beneficial [3]. Here, we report a case of rapidly progressive glomerulonephritis associated with infective endocartitis in which the clinical symptoms were successfully improved by the treatment with short-term steroid therapy.

## Case Presentation

A 24-year old man was admitted to our hospital because of macrohematuria and general malaise, along with insidious deterioration of renal function. The patient had been diagnosed as having ventricular septum defect (VSD) without complications and had been well six months before admission, when the patient presented a temperature of 38.0°C and a productive cough. One month before admission, the patient was admitted elsewhere because of fever, general malaise, and macrohematuria. The temperature was 38.6°C, the pulse was 120 beats per minute, and respirations were 24 times per minute. The blood pressure was 130/58 mmHg. On physical examination, the patient appeared acutely ill and pansystolic murmur of Levine III/IV was noted at the 4th left sternal border (LSB). The urine was positive for hematuria (+++) and protein (+++); the sediment contained 150–160 RBC/hpf and 10–15 granular casts/hpf. Laboratory test revealed 2.04 mg/dl of serum creatinine, 33.3 mg/dl of BUN, and 9.0 g/dl of hemoglobin. Echocardiogram demonstrated VSD and vegetation on the tricuspid valve associated with regurgitation. The patient was transferred to our hospital for evaluation of renal dysfunction.

**Figure 1:**
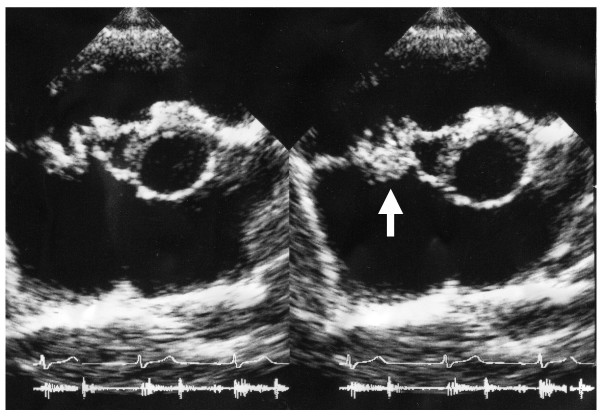
**Vegetation on tricuspid valve by echocardiography. **Arrow denotes the vegetation.

**Figure 2:**
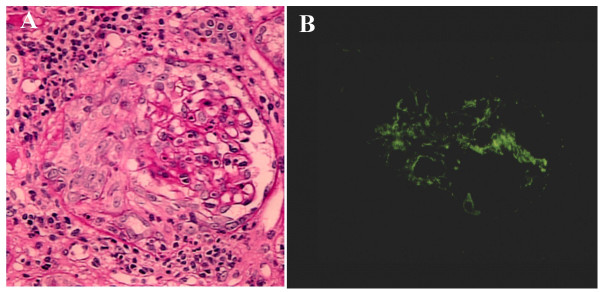
**Crescentic glomerulonephritis induced by infective carditis on PAS staining and IF. **A. PAS staining demonstrated circumferential and cellular crescent formation with interstitial nephritis. B. IF demonstrated C3 positive staining in mesangial area.

On the first hospital day, the temperature was 39.4°C and the physical examination demonstrated pansystolic murmur at the 4th LSB again, but no lymphoadenopathy and no localizing signs for a focus of infection. Laboratory findings were: anemia (Hb 8.6 g/dl, Ht 26.5%), presence of d-dimer (10.3 mg/ml), BUN 41 mg/dl, serum creatinine 2.49 mg/dl, proteinuria 0.96 g/24 h, 24 h Ccr 30.0 ml/min, hypocomplementemia (C3 29 mg/dl, CH50 16.5 U/ml), positive for inflammatory sign (CRP 2.8 mg/dl, ESR 104 mm/h), and positive for cryoglobulinemia (Table 1). Antibodies for DNA, RNP, Sm, and myeloperoxidase and proteinase 3 ANCA were normal (Table 1). Echocardiogram again showed vegetation on tricuspid valve and TR (Figure 1), suggesting the presence of right-sided bacterial endocarditis. However, there were no histories of tooth extraction, skin injury, or drug addiction and serology for viruses including HIV-1 was negative. Specimens of blood were obtained 5 times for culture and demonstrated Propionibacterium Acnes. A renal biopsy specimen showed typical crescentic glomerulonephritis (Figure 2A) with interstitial inflammatory cell infiltration on PAS staining. Ten of eighteen glomeruli had cellular crescents without fibrocellular and fibrous crescents. Immune reactants such as C3 (Figure 2B) and Ig M were found in peripheral capillary walls and in the mesangium.

**Table 1:** Laboratory Values on Admission

Hematocrit (%)	26.5	IgG (mg/dl)	2707
Hemoglobin (g/dl)	8.6	IgM (mg/dl)	768
White cells (per mm^3^)	6100	IgA (mg/dl)	449
Total protein (g/dl)	6.8	C3 (mg/dl)	29
Total bilirubin (mg/dl)	0.3	C4 (mg/dl)	35
Aspartate aminotransferase (U/liter)	29	Rheumatoid factor (U/ml)	483
Alanine aminotransferase (U/liter)	29	Anti-dsDNA (U/ml)	negative
Lactate dehydrogenase (U/liter)	666	Anti-GBM (U/ml)	negative
Alkaline phosphatase (U/liter)	75	MPO-ANCA titer	negative
Creatine phosphokinase (U/liter)	34		
Urea nitrogen (mg/dl)	41		
Creatinine (mg/dl)	2.49	Urinary sediment	
Sodium (mmol/liter)	137	Erythrocytes	+++
Potassium (mmol/liter)	4.7	Leukocytes	-
Calcium (mg/dl)	8.1	Cylinders	+
C-reactive protein (mg/dl)	2.8		

**Figure 3:**
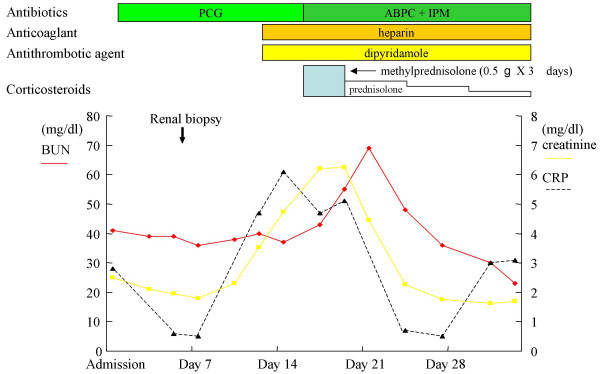
**Clinical course. **PCG; penicillin G, ABPC; ampicillin, IPM; imipenem

Bacterial endocarditis complicated with rapidly progressive glomerulonephritis was diagnosed and the intensive antibiotic therapy with penicillin G was started, without clinical improvement of renal function (on day 7 serum creatinine 6.21 mg/dl) or resolution of fever over the next 7 days. The initial antibiotics were replaced with ampicillin and imipenem in addition to low dose of corticosteroid treatment initiated with intravenous methylprednisolone 0.5-g per day for 3 consecutive days followed by oral prednisolone 30-mg, 20-mg, and 10-mg per day each for 3 days, respectively. On day 16, laboratory test demonstrated normalization of inflammatory signs (CRP 0.5, ESR 11 mm/h), serum complements, and circulating immune complex and negative for blood culture and cryoglobulin. After the short-term treatment of low dose of corticosteroid combined with continuous antibiotics, high fever and renal insufficiency were dramatically improved (Figure 3). The antibiotic therapy lasted for 40 days until the day of the tricuspid valve-plasty and VSD closure. The patient was finally able to undergo the surgical operation, resulting in successful recovery from endocarditis-induced rapidly progressive glomerulonephritis. He is now well and laboratory test showed normal serum creatinine 0.72 mg/dl, while echocardiogram demonstrated mild regurgitation of tricuspid valve.

## Discussion

Therapy with a bactericidal antimicrobial agent or combination of agents is usually effective [1-3], although in some cases antibiotic therapy fails, resulting in end-stage renal failure requiring dialysis therapy. Here, we present a patient complicated with VSD who developed rapidly progressive glomerulonephritis accompanying right sided-subacute bacterial endocarditis caused by Propionibacterium acnes. Although Propionibacteium acnes is considered to be contaminant, it has been found to be a pathogen of infective endocarditis in spite of its weak virulence [4]. Furthermore, case-reports of shunt nephritis associated with Propionibacterium acnes were also reported [5-7]. Membranoproliferative glomerulonephritis is the lesion most frequently seen in shunt nephritis, but in some patients in whom untreated and inadequately treated bacteremia persists, mild renal involvement may progress to the development of severe impairment such as crescents and sclerotic glomeruli, possibly through the prolonged immune-mediated pathogenesis [8]. In the present case, the prolonged exposure to the weak pathogen resulted in the development of crescentic glomerulonephritis in association with circulating immune complexes and cryoglobulinemia. Moreover, in the present case, the antibiotic therapy alone was only able to suppress circulating bacteremia, but failed to decrease the size of vegetation and the nest of bacteria. However, the clinical improvement of our case was thought to be a delayed response to continued antibiotic therapy and the addition of anticoaglants [3,9,10]. Some case reports also showed that immunosuppressive therapies such as plasmapheresis, cyclophosphamide, and azathioprine with antibiotics could recover renal dysfunction of infective endocarditis-induced crescentic glomerulonephritis [3,11]. Recently, a short-tem and low dose of anti-inflammatory corticosteroid has also shown to be potentially effective in reducing the risk of death in patients with sepsis [12,13]. In conclusion, we suggest that patients with rapidly progressive glomerulonephritis associated with infective endocarditis might be treated with a short-term and low dose of corticosteroid successfully, in the case presenting the clinical and biological evidence of immune-mediated pathogenesis with the prolonged duration of the illness.

## Competing interests

The author(s) declare that they have no competing interests.

## Authors' contributions

Koya D and Shibuya K cared the patients, took the picture, and wrote the paper.

Haneda M and Kikkawa R discussed the case.

## Pre-publication history

The pre-publication history for this paper can be accessed here:


